# Social Environment Influences Performance in a Cognitive Task in Natural Variants of the *Foraging* Gene

**DOI:** 10.1371/journal.pone.0081272

**Published:** 2013-12-12

**Authors:** Nancy R. Kohn, Christopher J. Reaume, Celine Moreno, James G. Burns, Marla B. Sokolowski, Frederic Mery

**Affiliations:** 1 Laboratoire Evolution, Génome et Spéciation, CNRS, Gif sur Yvette, France; 2 Department of Ecology and Evolutionary Biology, University of Toronto, Toronto, Ontario, Canada; 3 Department of Biology, University of Missouri–Saint Louis, Saint Louis, Missouri, United States of America; Queensland Brain Institute, Australia

## Abstract

In *Drosophila melanogaster*, natural genetic variation in the *foraging* gene affects the foraging behaviour of larval and adult flies, larval reward learning, adult visual learning, and adult aversive training tasks. Sitters (*for* ^s^) are more sedentary and aggregate within food patches whereas rovers (*for^R^*) have greater movement within and between food patches, suggesting that these natural variants are likely to experience different social environments. We hypothesized that social context would differentially influence rover and sitter behaviour in a cognitive task. We measured adult rover and sitter performance in a classical olfactory training test in groups and alone. All flies were reared in groups, but fly training and testing were done alone and in groups. Sitters trained and tested in a group had significantly higher learning performances compared to sitters trained and tested alone. Rovers performed similarly when trained and tested alone and in a group. In other words, rovers learning ability is independent of group training and testing. This suggests that sitters may be more sensitive to the social context than rovers. These differences in learning performance can be altered by pharmacological manipulations of PKG activity levels, the *foraging (for)* gene's gene product. Learning and memory is also affected by the type of social interaction (being in a group of the same strain or in a group of a different strain) in rovers, but not in sitters. These results suggest that *for* mediates social learning and memory in *D. melanogaster*.

## Introduction

How social environment affects an individual's behaviour has recently received increased attention [1]. Individual differences in response to social interactions are widespread, including some extreme cases of social disorders in humans (e.g. autism and antisocial personality disorder). Understanding the genetic and neural contributions that lead to variations in response to social interactions can help us understand the molecular bases of social behaviour, how it is encoded in nervous systems, and how social behaviour has evolved. The fruit fly, *Drosophila melanogaster*, has emerged as a useful model for studying gene by social environment interactions [1]–[5]. Here we present evidence for natural genetic variation in response to social interactions.

Being in a group can change an individual's behaviour [6]. For example, in *Drosophila* courtship, the presence of a potential sexual partner modifies a fly's behaviour and induces a sequence of species-specific actions [7]. Aggression, another form of social interaction, can also change an individual's behaviour [8], [9]. The modulatory systems affecting this behaviour, primarily octopamine and serotonin, have begun to be described [10]–[12]. By definition, courtship and aggression can only be analysed in a social environment since their execution requires at least two individuals. Social environment also affects other behaviours that were first considered to be non-social. For example, an individual's circadian activity and the expression of its clock genes (e.g. *period*, *timeless*, and *clock*) are modified by the presence of other flies and the group composition has significant effects on pheromonal output of the clock [2], [13], [14]. While social interactions can modify non-social behaviours, the reverse is also true. For example, manipulation of a flies' ability to smell can alter their social interaction [15]. Therefore, fruit flies are likely to acquire information while in a group and the social environment can potentially alter their behaviour.

Social context can also impact fly learning (see [1] for review). In most classical olfactory learning paradigms, *Drosophila* are trained in groups to associate an odour with an untrained stimulus (positive or negative) and then tested in groups for the learned modification of odour preference. Previous studies suggested that each fly behaved independently [16]–[18]. However, recent findings challenged this view. Chabaud et al. [19] found a strong influence of social context on memory consolidation.

While the relationship between social interaction and learning has been demonstrated, the genetic component underlying such behaviour has not been investigated. This study investigates whether or not natural genetic variation could lead to differences in learning performance in a manipulated social environment. Naturally occurring allelic variation in the *foraging* (*for*) gene of *D. melanogaster* was studied. The *for* gene is involved in regulating a number of food-related behaviours in flies [20], and impacts learning and memory traits in larval [21] and adult flies [22]–[24] as well as fear conditioning in mice [25].

The *foraging* gene encodes a cGMP-dependent protein kinase (PKG), and natural allelic variation in the *D. melanogaster for* results in the ‘rover’ (*for^R^*) and ‘sitter’ (*for* ^s^) behavioural variants that differ in PKG activity levels [26]. Rover larvae [27] and adult heads [26] have higher PKG enzyme activity than sitters. Adult rovers leave food patches more readily [28]–[30], visit more food patches, and tend not to revisit food patches compared to sitter adults who tend to be more sedentary and aggregate within food patches [28], [30]–[33]. All of these movement-related parameters could have a profound effect on the frequency and type of social interactions the variants experience, and how they are used when learning about their environment. Given *for*'s role in rover/sitter movement patterns during foraging behaviour, we suggest that these natural variants are likely to experience different social environments.

Here we asked whether *for* influences social interactions in the context of associative learning. We hypothesized that adult rovers and sitters would perform differently in a classical olfactory training test, depending on whether they were trained and/or tested in a social environment. We show: 1) the acquisition of information is modulated by the presence or absence of other flies in sitters but not in rovers, and 2) the genetic composition (rover or sitter) of the group affects individual performance of naïve rovers but not naïve sitters.

## Materials and Methods

### (a) Fly strains

*D. melanogaster* rover (*for* ^R^) and sitter (*for* ^s^) natural allelic variants of the *foraging* (*for*) gene [28], [34] were used. Flies were cultured on standard medium and kept in groups. We bred, trained, and tested flies at 22±2°C.

### (b) Learning assay

We used an aversive Pavlovian olfactory training assay [35] that trained flies to associate one of two odours (3-Octanol: OCT or 4-methycyclohexanol: MCH, Sigma) with an aversive mechanical shock. For training in groups we used 50 adult fruit flies (3 to 5 days old, sexes mixed) per group. In each training cycle, we first exposed flies for 60 s to one odour coupled with a mechanical shock (2000 rpm vibration pulses of 1 s duration, delivered every 5 s). Flies were given a 60 s rest period (no odour and no shock) and then the second odour was delivered for 60 s without a mechanical shock. The training cycle ended with a second 60 s rest period. We trained flies with three consecutive training cycles. Half the flies were trained to associate OCT with the shock and the other half were trained to associate MCH with the shock. 5–10 min after training, we tested the flies by placing them at the choice point of a T-maze where they were exposed to two converging currents of air; one carrying OCT and the other MCH. We allowed the flies 120 s to choose between the two odours. We calculated a performance index (PI) by taking the difference between the proportion of flies choosing OCT when MCH was associated with the shock and the proportion of flies choosing OCT when OCT was associated with the shock.

The training and testing of individual flies followed the same procedures as with groups of flies. However, just before training or testing we separated out individual flies (via aspiration without anaesthesia) so that they were trained and/or tested alone in the T-maze. We previously found that aspiration and the transfer of flies had no effect on learning performance in both lines (Figure S1). Following previous studies [16], [18], [19], [36], [37], we determined individual PI by pooling the choices of six consecutive individuals and then calculating the difference between the proportion of flies choosing OCT when trained to avoid OCT or MCH. This global score represents a mean of the individuals' behaviour.

### (c) Pharmacological treatments

To better delineate the influence of PKG activity on social interactions, we administered two pharmacological agents known to affect PKG enzyme activity in adult heads [38]. We treated rover and sitters flies with both a PKG inhibitor and a PKG activator. Following Dawson-Scully et al. [38] the PKG inhibitor, KT5823 (Sigma), was delivered at 1 mM and the PKG activator, 8-Bromo-cGMP (Sigma), at 10 mM solubilised in dimethyl sulfoxide (DMSO). Ten adult flies were introduced into a standard fly culture vial containing 10 µl of the prepared drug-DMSO solution applied on a lab tissue that was crushed to the base of a vial and covered with a second tissue to prevent direct contact of the flies to the drug solution. The tubes were capped with a sponge and covered with a fingertip of a latex glove to prevent the volatilized drugs from escaping. We then let the flies incubate in the dark for one hour. Control flies were given DMSO without the PKG activator or inhibitor. Immediately afterwards, we trained and tested individual flies as described above.

### (d) Statistical analysis

PIs were analysed using ANOVAs and included, depending on the experiment, the strain (rover or sitter) of the focal fly, the social context (alone or in a group), the group composition (group of rovers or sitters) and the pharmacological treatment as factors. For statistical comparisons of the PI (but not for graphical representations of the data) we arc-sine-square-root-transformed the proportions and checked for normal distributions of the data before analyses [39]. We performed post hoc comparisons between treatments when significant differences among treatments were found. For experiment 5, we conducted t-tests comparisons to test whether individual naïve flies placed into groups of trained flies performed significantly better than chance.

## Experiment 1: The Effect of Being in a Group and Pkg Activity

We first tested whether learning performance was affected by rover or sitter flies being alone or in groups. Using the protocol described above, groups and individual flies were trained and tested. Afterward, we calculated and compared their PI. We then tested individual rovers and sitters using the pharmacological treatments above to delineate the role of PKG activity.

### (a) Results

**Figure 1:**
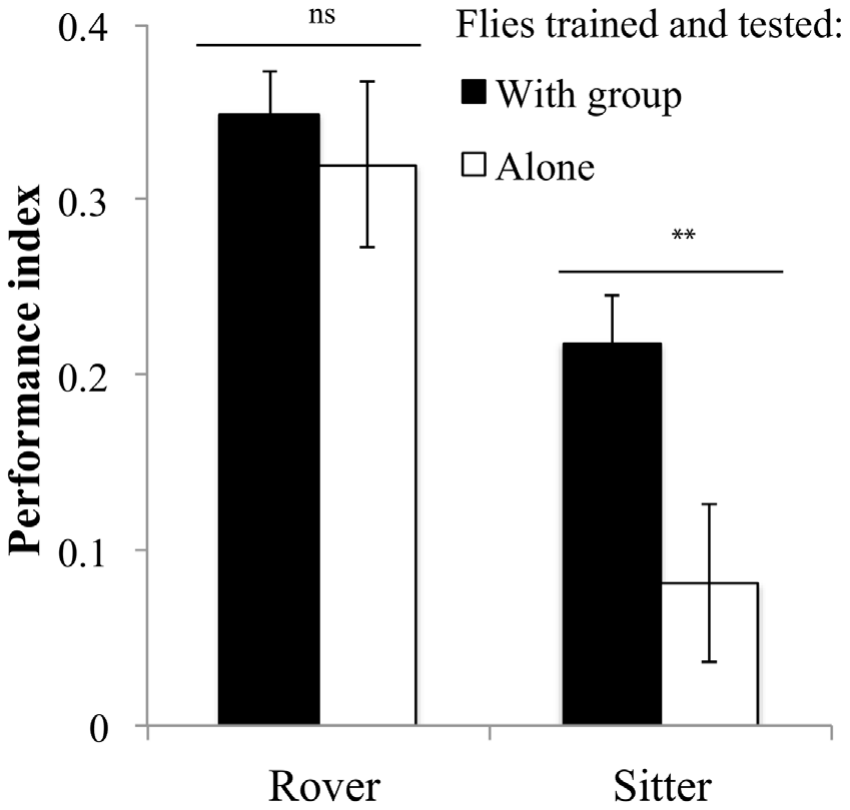
Performance of rover and sitter adult *D. melanogaster* when trained and tested in groups or individually. Rover: group: PI = 0.34±0.03 N = 52 PI; alone: PI = 0.32±0.04 N = 34 PI; Sitter: group: PI = 0.21±0.02 N = 52 PI; alone: PI = 0.08±0.04 N = 34. Error bars represent ±SEM. Comparison between treatments. ns: P>0.05; *: P<0.05; **: P<0.01; ***: P<0.001.

Similar to previous research on STM [24], [40], when trained and tested in groups, rovers had significantly higher PI than sitters (Figure 1, *F_1,102_* = 13.02*p*<10^−3^). When trained and tested alone, rovers performed as well as when they were trained and tested in groups (*F_1,88_* = 0.34 *P* = 0.55). However, individual sitters had significantly lower PI than groups of sitters (*F_1,89_* = 7.6 *P* = 0.007; Figure 1).

**Figure 2:**
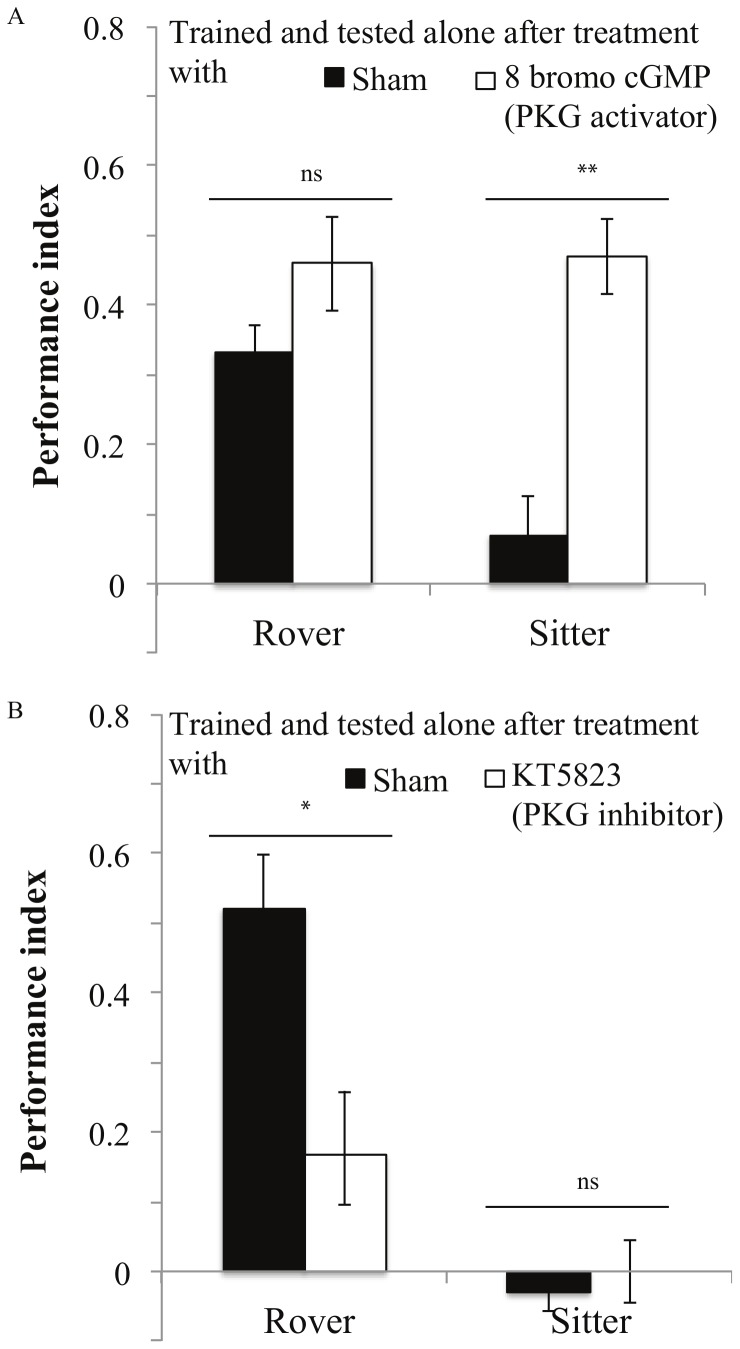
Performance of rovers and sitters when trained and tested alone with or without a pharmacological treatment. **A:** Treatment with the PKG activator 8-bromo-cGMP. Rover: DMSO: PI = 0.33±0.1 N = 11 PI; DMSO+8 bromo: PI = 0.45±0.09 N = 9 PI. Sitter: DMSO: PI = 0.07±0.08 N = 12 PI; DMSO+8 bromo: PI = 0.46±0.08 N = 11 PI **B:** Treatment with the PKG inhibitor KT5823: Rover: DMSO: PI = 0.52±0.1 N = 8 PI; DMSO+KT5823: PI = 0.16±0.12 N = 8 PI. Sitter: DMSO: PI = 0.02±0.04 N = 11 PI; DMSO+KT5823: PI = 0±0.08 N = 11 PI. Error bars represent ±SEM. Comparison between treatments. ns: P>0.05; *: P<0.05; **: P<0.01; ***: P<0.001.

Manipulations of PKG levels impacted the PI of individuals. The PKG activator (8-bromo-cGMP) significantly improved the PI of sitters (Figure 2A, *for^s^*: *F_1,21_* = 11.5, *P* = 0.003). However, rover performance was not affected by the PKG activator (Figure 2A, *for^R^*: *F_1,18_* = 0.763, *P* = 0.39). Congruently, when treated with the PKG inhibitor (KT5823), individual rovers had significantly lower PI than sham control rover flies (Figure 2B, *for^R^*: *F_1,14_* = 4.7, *P* = 0.04). As expected, sitter response was not affected by the PKG inhibitor (Figure 2B, *for* ^s^: *F_1,17_* = 0.2, *P* = 0.6)

These results suggest that social environment affects the learning performance of rovers and sitters differently. Rovers show similar responses when in a group or alone whereas sitters respond differently depending on the social environment. Being in a group facilitates sitters' learning performance.

## Experiment 2: Variation in the Social Context between Training and Testing

Sitters trained and tested in a group display a significantly greater PI than sitters trained and tested alone. However, this observation does not indicate which phase(s), the training phase, the testing phase, or both phases together, are affected by the group setting the most. Do sitters and rovers trained and tested in a group display greater improved learning performance compared to those with the group setting in only one of these phases? To decipher which phase(s) best improves learning performance, three training-testing conditions were implemented. In one condition, rover and sitter flies were trained alone and then tested in a group. In another condition, flies were trained in a group and then tested alone. A control condition was also used where all flies were trained and tested in a group. When tested in a group, the focal individual fly had its wings clipped for identification purposes. We placed groups of flies on ice and clipped the tips of their wings twenty-four hours before training. Under these conditions, wing clipping had no effect on the PI or the response to social interactions (Figure S1) [19]. We individually trained wing-clipped flies and then introduced them into a group of trained flies of the same genotype prior to testing. After testing, we were able to determine which arm of the T-maze the wing-clipped fly was in. For flies that were trained in a group and then tested alone, we aspirated out individual flies from the groups and then tested them alone in the T-maze. The PI for individual focal flies were compared to the PI of control flies trained and tested in groups.

### (a) Results

**Figure 3:**
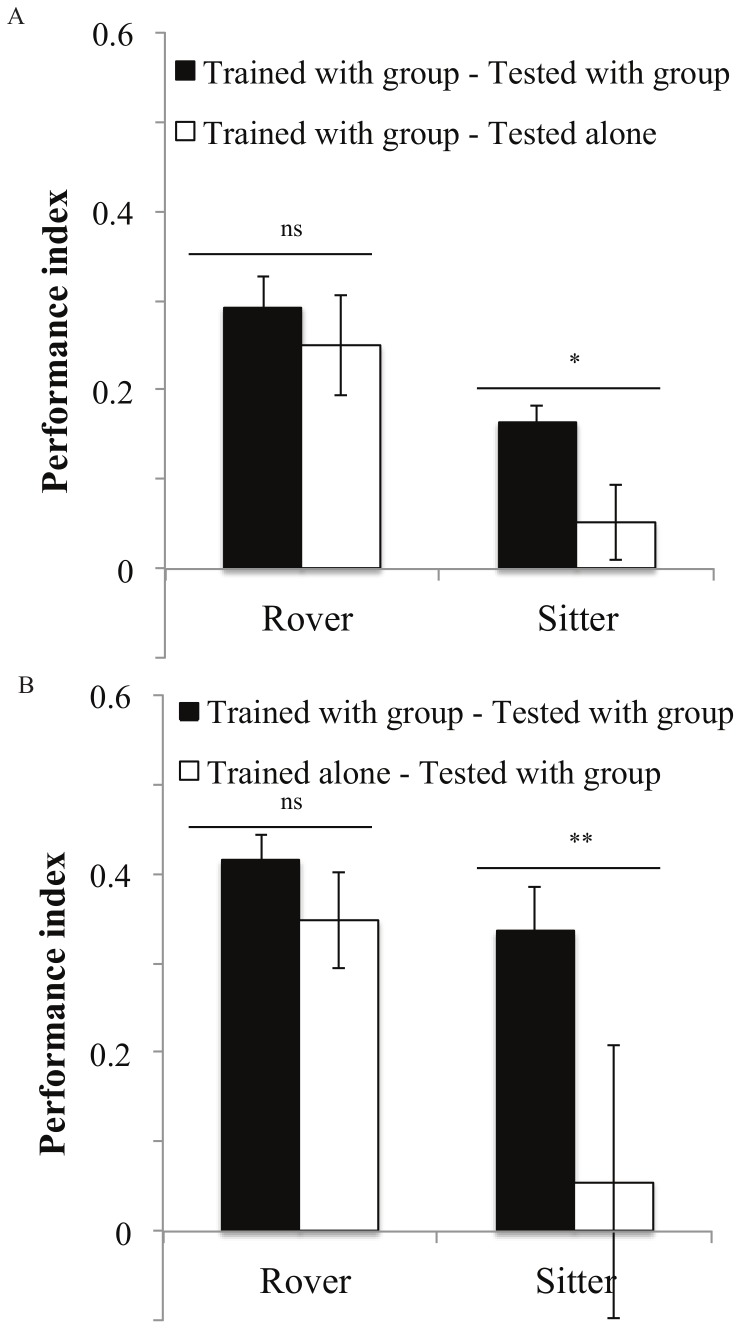
Performance of rovers and sitters when the training and testing were performed under different social contexts. **A:** flies were trained in groups and then either tested in groups of the same genotype or individually. Rover: group: PI = 0.3±0.03 N = 23 PI; alone: PI = 0.25±0.05 N = 12 PI. Sitter: group: PI = 0.16±0.01 N = 24 PI; alone: PI = 0.05±0.04 N = 16 PI. **B:** flies were trained individually or in groups and then tested within a trained group of the same genotype. Rover: group: PI = 0.41±0.03 N = 25 PI; alone: PI = 0.34±0.05 N = 7 PI. Sitter: group: PI = 0.33±0.05 N = 21 PI; alone: PI = 0.05±0.15 N = 6 PI. Error bars represent ±SEM. Comparison between treatments. ns: P>0.05; *: P<0.05; **: P<0.01; ***: P<0.001.

Sitters had significantly lower PI when trained and tested under different social contexts (group training-testing vs. trained in a group-tested alone: *F_1,38_* = 7.27, *P* = 0.01; group training-testing vs. trained alone-tested in a group of trained sitters: *F_1,48_* = 12.19, *P* = 0.001; Figure 3A and 3B). In contrast, rovers showed no significant differences in PI under these conditions (group training-testing vs. trained in a group-tested alone: *F_1,33_* = 0.47, *P* = 0.49; group training-testing vs. trained alone-tested in a group of trained rovers: *F_1,46_* = 1.57, *P* = 0.21; Figure 3A and 3B).

## Experiment 3: Effect of Group Composition on an Individual

Sitter flies seem particularly affected by the social context and we could not disentangle the effects of the training and the testing phase. On the other hand, rover flies performed equally well and were not affected by the social context of training and testing. We next wondered whether the composition of the group itself could affect individual performance during the test phase. We trained individual focal flies alone (wing clipped as previously described) and then tested them within groups of trained flies that were from the same or different strain. The focal fly and the group were trained to associate mechanical shock with the same odour (OCT or MCH).

### (a) Results

**Figure 4:**
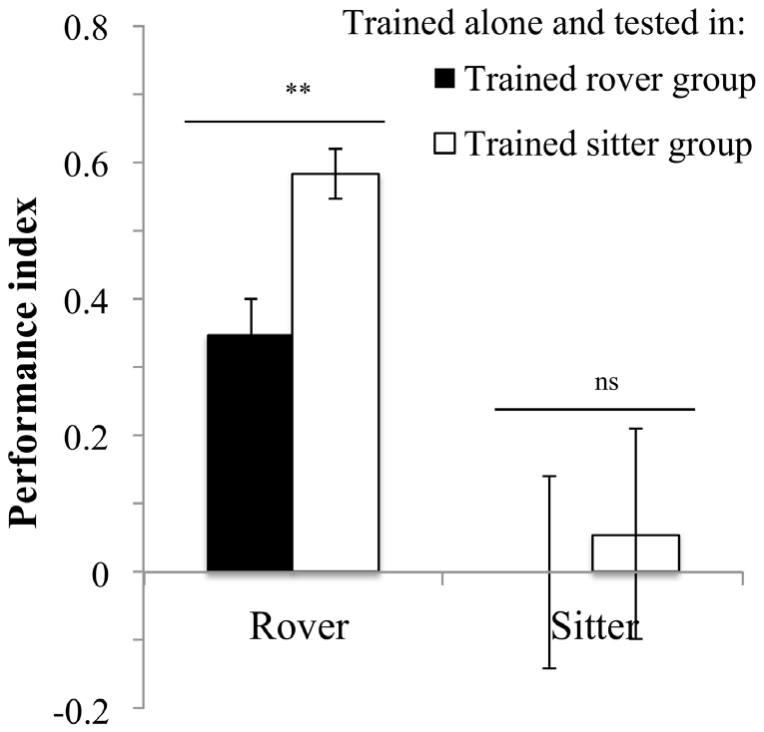
Effects of group composition during testing. Flies were individually trained and then tested within a trained group of the same strain or a different strain. Rover: in rover: PI = 0.34±0.05 N = 7 PI; in sitter: PI = 0.58±0.03 N = 6 PI. Sitter: in sitter: PI = 0.05±0.15 N = 6 PI; in rover: PI = 0±0.14 N = 7 PI. Error bars represent ±SEM. Comparison between treatments. ns: P>0.05; *: P<0.05; **: P<0.01; ***: P<0.001.

Rovers trained alone performed differently depending on the group composition during the test phase. Rover individuals had significantly lower PI when tested in a group of trained rovers than when tested in a group of trained sitters (Figure 4, *F_1,11_* = 12.2, *P* = 0.005). Sitter performance when trained alone was not affected by group composition and, in accordance with experiment 2, showed no response to training (Figure 4, *F_1,11_* = 0.07, *P* = 0.79).

## Experiment 4: Effect of Group Experience on an Individual

It is unclear why rovers in experiment 3 were affected by the composition of the group. We hypothesized that the difference in rover behaviour can be attributed to the role of personal information (provided by training alone) versus social information (provided by the group during testing) in this system. In a recent study, Foucaud et al. [41] observed variation between rover and sitter lines in their reliance on personal versus public information in a spatial learning task. Focal flies were first individually trained and then introduced into a group of naïve flies of the same or other strain. The choices of the individual flies indicated whether they used personal information (i.e. make the proper choice according to their own training protocol) or social information (i.e. make choices according to the behaviour of the naïve group).

### (a) Results

**Figure 5:**
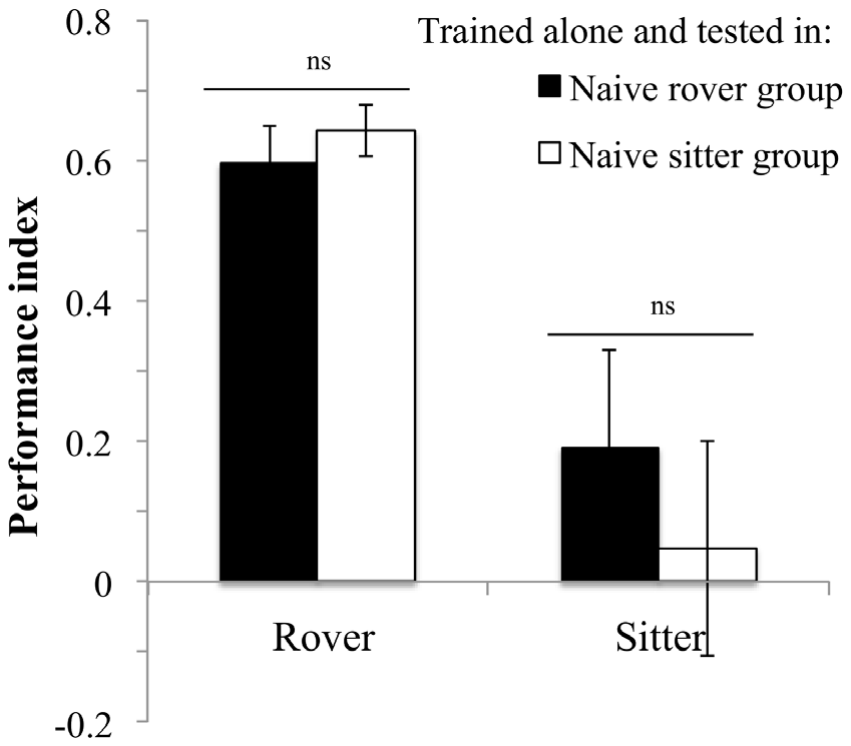
Performance of individually trained flies when tested within a group of naive rovers or naïve sitters. Rover: naive rover group: PI = 0.59±0.04 N = 7 PI; naive sitter group: PI = 0.64±0.06 N = 7 PI. Sitter: naive rover group: PI = 0.19±0.09 N = 7 PI; naive sitter group: PI = 0.04±0.08 N = 7 PI. Error bars represent ±SEM. Comparison between treatments. ns: P>0.05; *: P<0.05; **: P<0.01; ***: P<0.001.

The performance of rovers that were trained alone was not affected by naïve groups of rovers or sitters during testing (Figure 5, *F_1,11_* = 0.32, *P* = 0.58), indicating that personal information was weighted more heavily than public information in this context. As expected, sitters that were trained alone showed no response to the training phase when tested with either naïve rover or sitter groups (Figure 5, *F_1,12_* = 1.17, *P* = 0.3).

## Experiment 5: Naïve Individuals Tested in a Trained Group

Although fly performance is not affected by personal versus social information in experiment 4 where the focal fly is trained, it is not clear whether the same would hold true if the focal fly is naïve (untrained). We asked whether group composition affects an individual's behaviour when only public information was available during the test. Groups of rovers or sitters were trained as above, and just before placing them into the T-maze we added a focal naïve fly (not trained and wings clipped) of the specified strain. After testing, we determined which arm of the T-maze the focal fly was in. We calculated the PI of the focal naïve flies the same way as their corresponding trained flies. Note that, under these circumstances, significant positive PI indicates that naïve flies are using public information produced by the trained members of their group. In order to better understand the role of PKG encoded by the *foraging* gene in this specific task, we pharmacologically treated naïve individuals and tested them in a trained group. We delivered the PKG inhibitor, as in the pharmacological treatments above, to naïve rover individuals and the PKG activator to naïve sitter individuals prior to introducing them into groups of trained sitters. Control naïve rover and sitter individuals were exposed to just DMSO. After testing we determined which arm of the T-maze the focal flies were in.

### (a) Results

**Figure 6:**
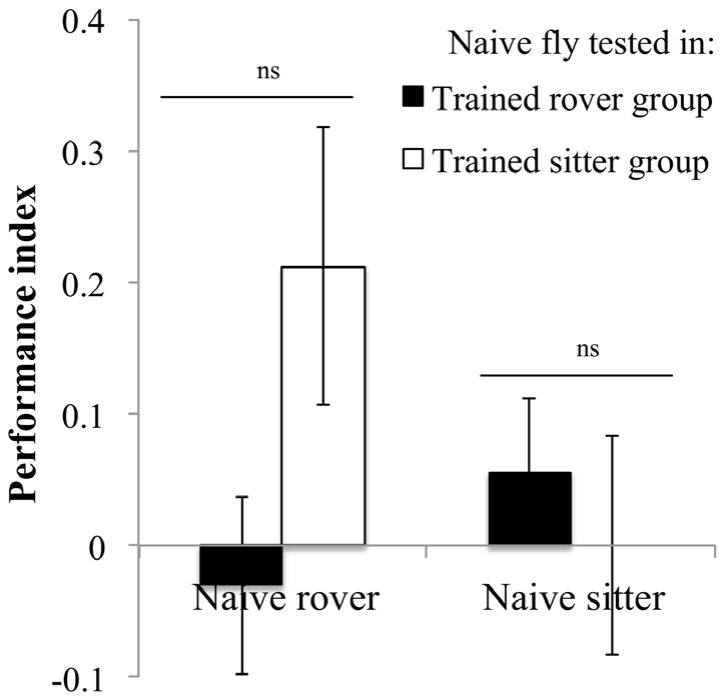
Performance of individual naïve flies tested in groups of conditioned flies. Positive PI indicates that the focal naive fly avoided the odour that the group was trained to avoid. Rover: rover group: PI = −0.03±0.1 N = 12 PI; sitter group: PI = 0.21±0.07 N = 12 PI. Sitter: rover group: PI = 0.05±0.08 N = 12 PI; sitter group: PI = 0±0.07 N = 12 PI. Error bars represent ±SEM. Comparison between treatments. ns: P>0.05; *: P<0.05; **: P<0.01; ***: P<0.001.

Trained groups had little impact on the choices of individual naïve sitters that randomly entered one or the other arm of the T-maze (Figure 6, comparison rover vs. sitter trained group *F_1,22_* = 0.24, *P* = 0.62). However, individual naïve rovers tended to behave differently depending on whether they were introduced into a group of trained rovers or sitters (Figure 6, trained group: *F_1,21_* = 3.64, *P* = 0.07). During the test, individual naïve rovers had a PI greater than random chance (PI = 0), when placed into groups of trained sitters (*t* = 2.97, *P* = 0.014), indicating that naïve rovers follow trained sitters. When individual naïve rovers were placed into groups of trained rovers their PI was not significantly different from random chance (*t* = −0.28, *P* = 0.7).

**Figure 7:**
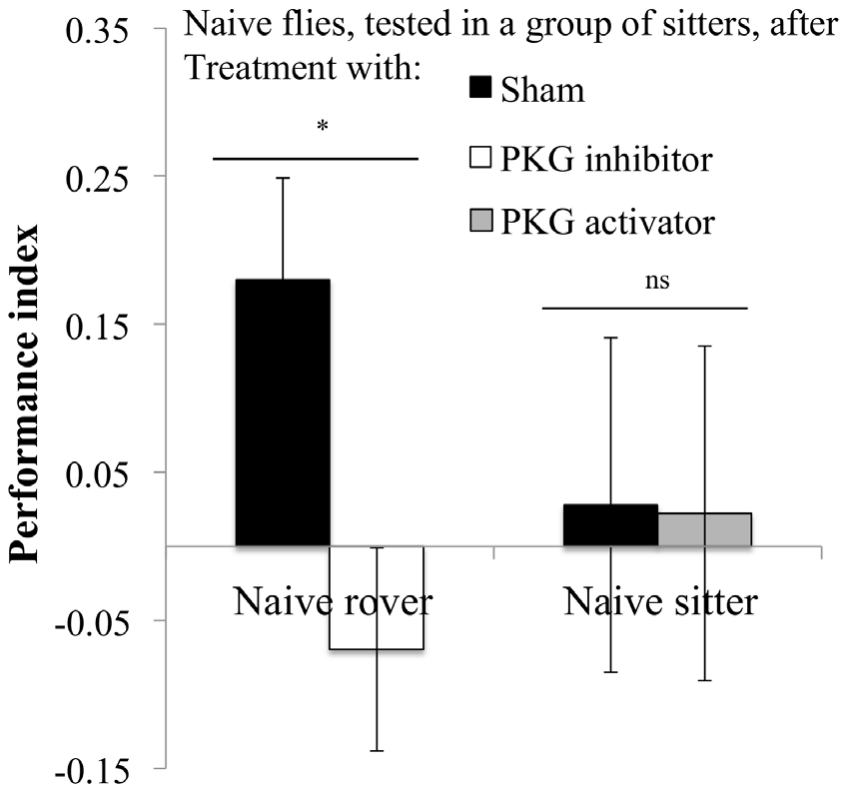
Performance of individual naïve flies after pharmacological manipulation when placed into groups of conditioned flies during the test. Rover: Sham: PI = −0.18±0.06 N = 11 PI; PKG inhibitor: PI = −0.07±0.07 N = 11 PI. Sitter: Sham: PI = 0.02±0.11 N = 9 PI; PKG activator: PI = 0.02±0.08 N = 12 PI. Error bars represent ±SEM. Comparison between treatments. ns: P>0.05; *: P<0.05; **: P<0.01; ***: P<0.001.

When we manipulated the PKG levels of naïve flies we found that the PKG inhibitor, KT5823, as expected, caused individual naïve rovers to decrease their PI in the presence of trained sitters groups, compared to sham controls (Figure 7, *F_1,20_* = 6.67, *P* = 0.018). However, the PKG activator, 8-bromo cGMP, did not induce an increase in the PI of sitters (Figure 7, *F_1,16_* = 0.004, *P* = 0.95).

## Discussion

We found that associative learning in *D. melanogaster* was influenced by social context and that this effect could be modulated by natural variation in the *foraging* gene which encodes PKG. The sitters, with lower PKG, when trained and tested alone, had significantly lower PI than sitters who were trained and tested in groups. Group versus individual training and testing did not affect the PI of rovers. Also, a change in social context between training and testing affected sitter but not rover performance. That is, sitter performance was significantly lower when trained in a group and then tested alone (or vice-versa). Our research not only extends previous work showing that social experience affects memory retrieval in *D. melanogaster* [19], but also demonstrates within-species variation in these effects.

To better understand how PKG impacts the effects of social context, we pharmacologically manipulated PKG levels in individual flies to ‘transform’ rovers into sitters, and sitters into rovers [38]. We found that PKG modulated associative learning in a social context. High PKG levels appear to enhance learning in a solitary context, as sitter performance was improved when PKG activity was increased. In contrast, pharmacologically lowering PKG activity decreased rover performance. This indicates that PKG activity levels can buffer the lack of learning performance found when sitters are alone.

The results suggest that sitter variants are more sensitive to social context than rovers. Given what is known about differences in sitter and rover movement patterns during foraging, this finding may not be surprising. As described above, rovers visit more patches, travel greater distances, and tend not to revisit previous patches compared to sitters [28]–[32]. Therefore, sitters, which tend to be more gregarious, may experience less variation in social interactions. Social isolation—particularly in a learning context—could thus constitute distractions or stresses to sitters.

The results also suggest that, after training, rovers use personal information over public information. Their PI was not diminished by the potentially distracting presence of naïve flies during testing. Surprisingly, the PI of individual rovers was also greater when tested in a group of trained sitters compared to a group of trained rovers. Learning and memory are known to be facilitated or impaired by the general level of stress [42]. In *Drosophila*, mechanical shocks induce the release of ‘Drosophila stress odorant’ (mainly CO_2_ [43]). Variation between rover and sitter strains in the release of or sensitivity to stress odorants could potentially affect their performance in the t-maze. This potential form of context-dependent social facilitation requires future investigation.

Interestingly, when only public information was available (experiment 5) rover flies tended to use this information whereas sitter flies did not. Naïve rovers introduced into a group of trained sitters had a positive PI, suggesting that they followed trained sitters. Naïve rovers introduced into a group of trained rovers did not have a positive PI. Sitters, however, did not follow trained rovers or trained sitters. This rover behaviour could indicate a type of sharing of ‘public information’ from sitters. Information sharing has been observed in *D. melanogaster* courtship behaviour [4]. It is important to note that trained rovers did not follow trained or naïve sitters, so the effect depends on the state of the individual rover. It may be that rovers use a strategy of ‘copying when uncertain’ [44].

In nature, *D. melanogaster* adults aggregate at food sources where a number of social behaviours take place including feeding, courtship, mating, and oviposition [45]. Previous research on flies suggests that the complexity of food search behaviour is not an individual process but requires social interactions between individuals [46], [47]. For example, social interactions between flies impacts what specific food patches are chosen [46]. First, ‘primer flies’ randomly search the environment and land on different food patches. Groups of follower flies then aggregate on the most favourable patches of food based on social interactions [46]. Several learning mutants (e.g., *dunce* and *rutabaga*) have been shown to have considerable effects on these complex search-aggregation social interactions involved in food search behaviour [46]. This suggests a possible overlap in the signalling pathways of the decision making processes involved in food search behaviour and those involved in learning.

More recent investigations suggest that a nitric oxide (NO) signalling pathway may also be involved in social interactions related to search-aggregation behaviour [47]. Mutants of soluble-guanylatecyclase (sGC) showed marked defects in a number of the search-aggregation behaviours including a decreased performance in exploratory aggregation, a disconnect of exploration from odour and taste cues, and a lack of preference for aggregated food patches [47]. Similarly, our results found that PKG signalling is important for performance in a decision making task where learning and memory are important. Pharmacological treatments showed that individual flies deficient in PKG were unable to perform in a learning task. Whether these two behavioural paradigms implicating NO and PKG signalling in social decision-making tasks are indicative of a similar underlying mechanism of signalling and sensory integration remains to be determined.

In the ant, *Phidole pallidudal*, social experience is known to effect brain PKG enzyme activity [48]. In honey bees, *foraging* gene RNA expression and PKG activity differs depending on a bees role in the hive which is socially determined [49] PKG is known to both phosphorylate proteins and act transcriptionally [20]. The present study provides further evidence for a conserved role for PKG in social functioning [1], [50]. The molecular and cellular mechanisms by which social context acts to affect PKG remains to be determined.

We have found that social context affects learning, and that this in part can be modulated by natural variation in a single gene. Variation in the *foraging* gene can affect not only how flies learn, but also how social context influences that learning. Along with previous evidence on how *for* affects social roles in honeybees and ants, this study provides new evidence for a social role in *Drosophila* suggesting *for* is a candidate gene for the genetic conservation of social behaviour [41], [50]. Taken together, these results contribute to the burgeoning field of socially-influenced behaviour in *D. melanogaster*, and demonstrate the effectiveness of the fruit fly as a model for studies on social behaviour. Understanding how genes function in social contexts is fundamental to understanding how social behaviour operates, as well as how it evolved [51]

## Supporting Information

Figure S1 **Effect of wing clipping, aspiration and transfer on individual fly learning performance.** Groups of 50 flies from each of the *for^R^* or *for^S^* lines were first trained using the same protocol as described in the methods section. Half of the flies were trained to avoid Octanol and the other half were trained to avoid MCH. These groups were composed of either intact or wing-clipped flies. Following training, a single fly was aspirated from one group (intact or wing-clipped) and transferred into the other group. Each group was then tested. The choice of the wing-clipped fly within the intact fly group (or intact fly within the wing-clipped group) was recorded and PIs were calculated as described in the methods section. Bars represent the PI of intact or wing-clipped transferred flies. In both lines, transferred flies showed significant response to the training procedure during testing (*for^R^*: wing-clipped: PI = 0.43±0.13 N = 6 PI; intact: PI = 0.39±0.18 N = 6 PI. *for^S^*: wing-clipped: PI = 0.34±0.08 N = 6 PI; intact: PI = 0.27±0.07 N = 6 PI) and wing-clipping had no effect on fly learning performance (effect of wing-clipping: *for^R^*: *F_1,10_* = 0.03 P = 0.86; *for^S^* : *F_1,10_* = 0.36 P = 0.56).
(TIF)
