# Supplementary figures and images for: Social Environment Influences Performance in a Cognitive Task in Natural Variants of the Foraging Gene
Source: PLoS One. 2013 Dec 12;8(12):e81272. doi: 10.1371/journal.pone.0081272 (PMC3861308; doi:10.1371/journal.pone.0081272)

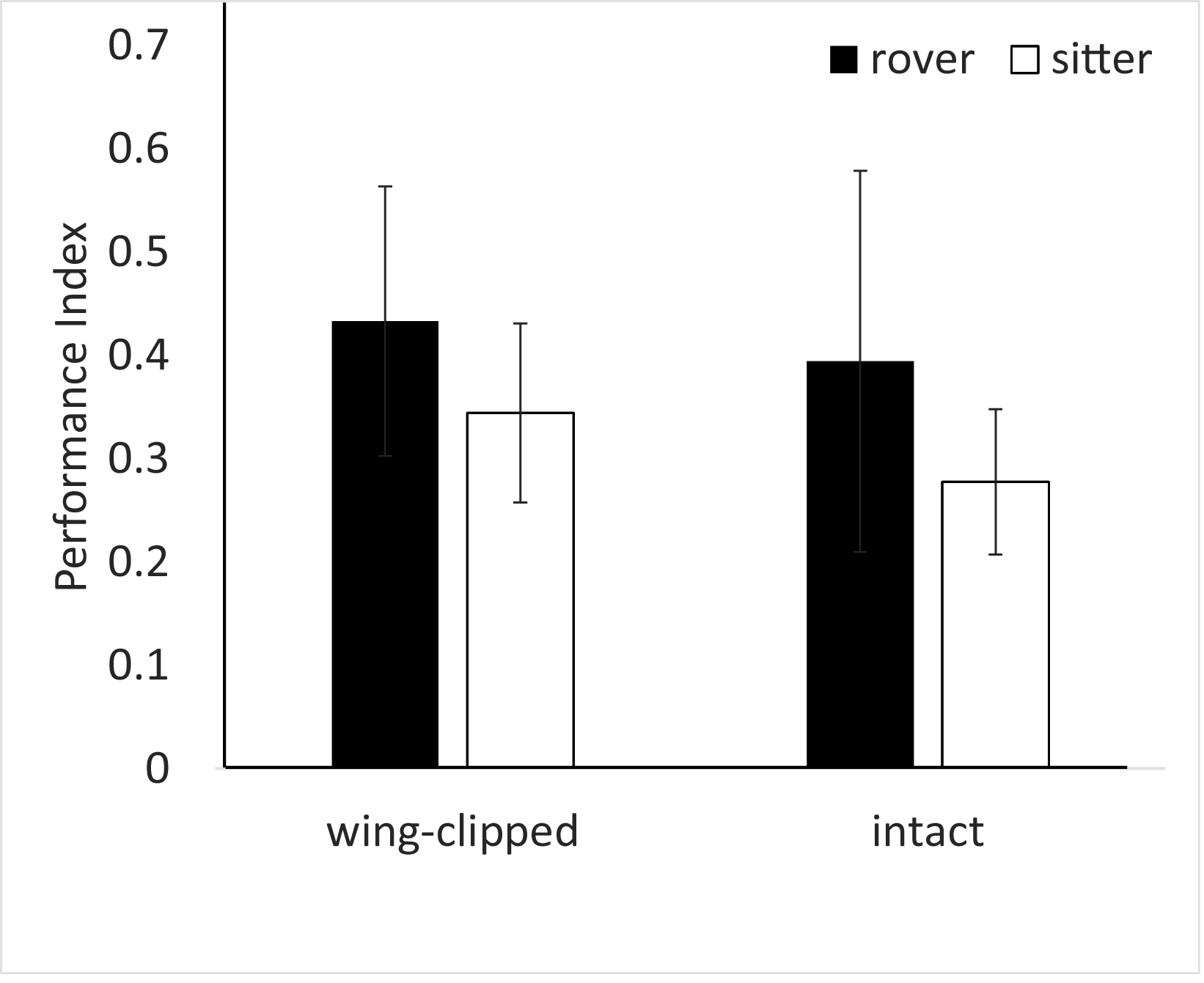

Supplement: Figure S1 — Effect of wing clipping, aspiration and transfer on individual fly learning performance. Groups of 50 flies from each of the forR or forS lines were first trained using the same protocol as described in the methods section. Half of the flies were trained to avoid Octanol and the other half were trained to avoid MCH. These groups were composed of either intact or wing-clipped flies. Following training, a single fly was aspirated from one group (intact or wing-clipped) and transferred into the other group. Each group was then tested. The choice of the wing-clipped fly within the intact fly group (or intact fly within the wing-clipped group) was recorded and PIs were calculated as described in the methods section. Bars represent the PI of intact or wing-clipped transferred flies. In both lines, transferred flies showed significant response to the training procedure during testing (forR: wing-clipped: PI = 0.43±0.13 N = 6 PI; intact: PI = 0.39±0.18 N = 6 PI. forS: wing-clipped: PI = 0.34±0.08 N = 6 PI; intact: PI = 0.27±0.07 N = 6 PI) and wing-clipping had no effect on fly learning performance (effect of wing-clipping: forR: F1,10 = 0.03 P = 0.86; forS : F1,10 = 0.36 P = 0.56). (TIF) [file pone.0081272.s001.tif]
